# Present, Future, and Novel Bioclimates of the San Francisco, California Region

**DOI:** 10.1371/journal.pone.0058450

**Published:** 2013-03-20

**Authors:** Alicia Torregrosa, Maxwell D. Taylor, Lorraine E. Flint, Alan L. Flint

**Affiliations:** 1 Western Geographic Science Center, United States Geological Survey, Menlo Park, California, United States of America; 2 Contractor, Western Geographic Science Center, United States Geological Survey, Menlo Park, California, United States of America; 3 California Water Science Center, United States Geological Survey, Sacramento, California, United States of America; University of Western Australia, Australia

## Abstract

Bioclimates are syntheses of climatic variables into biologically relevant categories that facilitate comparative studies of biotic responses to climate conditions. Isobioclimates, unique combinations of bioclimatic indices (continentality, ombrotype, and thermotype), were constructed for northern California coastal ranges based on the Rivas-Martinez worldwide bioclimatic classification system for the end of the 20^th^ century climatology (1971–2000) and end of the 21^st^ century climatology (2070–2099) using two models, Geophysical Fluid Dynamics Laboratory (GFDL) model and the Parallel Climate Model (PCM), under the medium-high A2 emission scenario. The digitally mapped results were used to 1) assess the relative redistribution of isobioclimates and their magnitude of change, 2) quantify the loss of isobioclimates into the future, 3) identify and locate novel isobioclimates projected to appear, and 4) explore compositional change in vegetation types among analog isobioclimate patches. This study used downscaled climate variables to map the isobioclimates at a fine spatial resolution −270 m grid cells. Common to both models of future climate was a large change in thermotype. Changes in ombrotype differed among the two models. The end of 20^th^ century climatology has 83 isobioclimates covering the 63,000 km^2^ study area. In both future projections 51 of those isobioclimates disappear over 40,000 km^2^. The ordination of vegetation-bioclimate relationships shows very strong correlation of Rivas-Martinez indices with vegetation distribution and composition. Comparisons of vegetation composition among analog patches suggest that vegetation change will be a local rearrangement of species already in place rather than one requiring long distance dispersal. The digitally mapped results facilitate comparison with other Mediterranean regions. Major remaining challenges include predicting vegetation composition of novel isobioclimates and developing metrics to compare differences in climate space.

## Introduction

Natural resource managers need tools to assess potential impacts of climate change across their local area of influence. Several approaches have been taken to use the outputs from global circulation models (GCM) to infer potential future change in vegetation, sea levels, and frequency of natural hazards such as wildfires and droughts. Selecting metrics from GCM outputs that facilitate quantitative comparison of biologically relevant changes is a major challenge for those seeking to understand the future effects of climate change on biological systems.

GCM climate variables are often analyzed as individual elements. For example, Cayan and colleagues [1] plot annual temperature anomalies from GCMs using the “A2” medium-high Intergovernmental Panel on Climate Change (IPCC) emission scenario to show marked shifts toward hotter annual temperatures in all future climate projections for California. Managers can use warming trend model convergence to support policy analysis based on a warming world. While raw climatic variables indicate potential magnitudes of change in one climate dimension, more integrated and biologically relevant combinations are better suited for exploring potential ecosystem response to changing climates and map out the salient differences among future climate projections.

Thompson and colleagues [2] introduced the use of orthogonal axes of temperature and precipitation at continental scales to analyze the climate space of various tree species in the present and thereby forecast potential distributions of these species under future climate projections. Many species-environment models have been developed using various modeling algorithms [3], [4], [5] and some, such as the climate envelope model BIOCLIM use as many as 35 different climate parameters in the form of independent, continuous variables along a gradient [6]. Forecasting regional vegetation change based on species-specific climate based models has been criticized. Most biotic communities undergoing change are comprised of large numbers of species across multiple taxa each with varying amounts of genetic amplitude that determine species response and adaptation to climate change. Modeling more than a few species is time consuming and non-climatic factors can be significant determinants of species distributions such as competition, predation, and dispersal. These are lacking from most models [7], [8] rendering their results incomplete. Forecasting onto future landscapes with climate envelopes that have no analogs to current conditions further complicates the task [9], [10].

An alternative approach is to work directly with bioclimates as categorical units and then populate these units with the species, communities, or functional traits distributed within these units. For example, several regions across the globe have a long term weather pattern of relatively mild year round temperatures, dry summers, and wet winters –a Mediterranean climate regime. Within this climate regime, an ombrotypic (wet/dry gradient) threshold distinguishes equivalent vegetation types. Those in more xeric conditions form a drought deciduous vegetation: coastal sage in California, garrigue in France, and renosterbos in South Africa; and in less xeric conditions a sclerophyllous evergreen shrub vegetation: chaparral in California, matorral in Chile, macchia in Italy, maquis in France and Israel, and fynbos in South Africa [11]. Focusing on bioclimate units has advanced research into the convergent physiognomy and ecophysiology of the regime [12], [13] as well as provided a better understanding of factors beyond climate that influence species response [14] and biodiversity [15], [16]. Likewise using a bioclimate framework at local scales, may help us think of innovative approaches to prepare for the unprecedented change that natural resource managers will be faced with.

The strong correspondence between vegetation and climate has long been used to map climate zones [17] and conversely to map vegetation [18], [19]. The landmark study of biologically relevant categorical breaks of climate variables by Rivas-Martinez [20], [21], [22] generated a hierarchical classification based on hundreds of thousands of relevés sampled along boreal - tropical latitudinal and elevational gradients. The Rivas-Martinez Worldwide Classification System (RMWBS) and other similar systems [23] provide numerically based methods whose spatial resolution is limited primarily by the resolution of the climate data. We use the RMWBS for our study area because it scales hierarchically and it captures with high sensitivity the precipitation and temperature patterns that differentiate plant communities in the Mediterranean climate [24], [25], [26] of the study area.

Downscaling GCM output to the local scale is particularly important for land management decisions that are implemented at the regional level such as land acquisition strategies to accommodate the dispersal of species of concern into more suitable habitat, restoration prioritization of one habitat patch over another, or managed translocation. Recognizing the challenge and the need for regional level climate change analysis and adaptation planning, the California Energy Commission (CEC) convened expert science panels to provide guidance, tools, and data to assist these efforts [27], [28]. An important guiding assumption for the CEC science panels was that even if future projections are uncertain, the use of the same projections would allow improved collaboration across adaptation management sectors such as energy, agriculture, water resources, and wildlife. The CEC white paper on climate scenarios for California [1] describes the GCM models and emission scenarios selected to investigate climate change in California. The CEC white paper on downscaling predictor variables [29] describes the two GCMs (PCM and GFDL) and two emission scenarios (A2, medium-high and B1, low) identified for cross-sector use. It also details the derivation of climate variables to drive regional and local scale models. The selection of GCM models, emission scenario and downscaling methods for this study are based on the results from the CEC science panel.

Traditionally the term macrobioclimate has been used for the five global bioclimate zones (tropical, Mediterranean, temperate, boreal, and polar). The term bioclimate has been used for the 5–7 categories within each macroclimate zone, and isobioclimate for the third nested combination of bioclimatic variants, thermotypes, and ombrotypes [see globalbioclimatics.org for more details]. We follow in this tradition for the products developed and discussed in this paper: 1) high resolution RMWBS isobioclimates for the end of the 20^th^ century, 2) RMWBS isobioclimates for the end of the 21^st^ century projected from two future climate models, PCM and GFDL; 3) change maps and transition matrices created by comparing 1 and 2; and 4) an alternative statistical approach for using bioclimate units to explore future plant community distribution.

The area of high resolution isobioclimates produced for this research coincides with the working boundary delineated by the Terrestrial Biodiversity and Climate Change Collaborative (TBC3), a group of university, government, and non-profit scientists conducting research and developing data sets for climate adaptation efforts in the greater San Francisco Bay Area [30]. This region is the largest biodiversity hotspot in the United States [31] based on the rarity-weighted richness index of rare and imperiled species of the United States [32]. Other studies currently underway by the authors use the same methodology and will expand the area of high resolution isobioclimate coverage to the entire State of California and beyond.

## Methods

**Figure 1:**
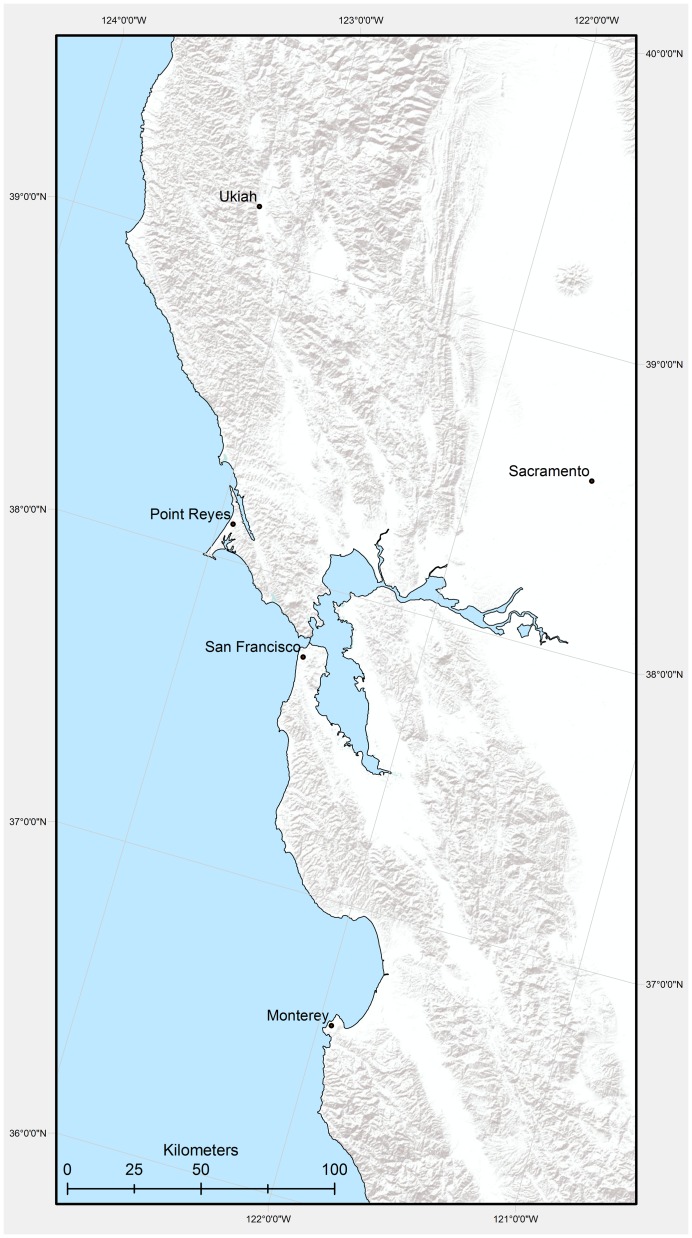
Topographic shaded relief of study area mapped in an Albers equal area projection.

The study area grid was defined as a gridded rectangle in a modified Albers equal area projection that included all 10 San Francisco Bay Area counties with an additional 100 km buffer north and south and a 30 km buffer to the east for connectivity studies. The rectangle represents six million hectares (62,304 square kilometers – 15 million acres) along the northern coast of California bounded by latitudes 39.55 and 36.29 north and longitudes 123.79 and 120.54 west (Fig. 1) with a grid cell resolution of 270×270 meters (854,651 cells).

### Rivas-Martinez Worldwide Bioclimatic Classification

The RMWBS integrates up to 26 climate parameters to derive and then segment into biologically relevant categories 3 primary indices, continentality, a measure of oceanic influence and temperature fluctuations; ombrotype, a measure of aridity; and thermotype, a synthetic measure of temperature regime. All calculations and mapping were done in the ARCGIS (geographic information system) [33]. The bay and delta areas were included in the analysis because bioclimate change also affects estuarine process of interest to resource managers in the region.

Each grid cell was categorized into isobioclimate types using the combined Rivas-Martinez bioclimatic indices of continentality [Ic], ombrotype [Io], and thermotype [Tmo] for two 30-year climatological periods, end of 20^th^ century (EO20^th^) and end of 21^st^ century (EO21^st^). Each of the three bioclimate indices is derived using the RMWBS climate parameter definitions, listed as equations in table 1, and the following RMWBS hierarchical classification procedures.

**Table 1:** Climate indices used to derive the continentality, ombrotype, and thermotype indices, in order of appearance in text.

Index	Description	Calculation	Units
Ic	Continentality	Ic = Tmax−Tmin	degrees Celsius
Io	Ombrotype	Io = Pp/Tp	ratio
Ios^2^	Ombrothermic index of the warmest bimonth of the summer quarter	[Ios_2_ = (Pps_2_/Tps_2_) 10]	scaled ratio
Ios^4^	Ombrothermic index of the summer quarter	[Ios_2_ = (Pps_4_/Tps_4_) 10]	
It	Thermicity	It = (T+m+M) 10	scaled (degrees Celsius)
Itc	Compensated Thermicity Index	if (18.0>Ic) then no compensation; if (18.0<Ic< = 21.0) then Itc = It+5; if (Ic>21.0) then Itc = It+((Ic -21)+15).	scaled (degrees Celsius)
m	Average Minimum temperature of the coldest month	Thirty year average of minimum temperatures for January	degrees Celsius
M	Average Maximum temperature of the coldest month	Thirty year average of maximum temperatures for January	degrees Celsius
Pp	Yearly Positive Precipitation	Total average precipitation of those months whose average temperature is higher than 0°C	mm
Pps_2_	Total precipitation of the warmest bimonth of the summer quarter	Thirty year average of the cumulative precipitation for July plus August	mm
Pps_4_	Total precipitation of the summer quarter	Thirty year average of the cumulative precipitation for May, June, July, and September	mm
T	Yearly Average Temperature	Thirty year average of the average annual temperature	degrees Celsius
Tmax	Average temperature of warmest month of the year	Thirty year average of July monthly average temperatures	degrees Celsius
Tmin	Average temperature of coldest month of the year	Thirty year average of January monthly average temperatures	degrees Celsius
Tp	Yearly Positive Temperature		degrees Celsius
Tp	Positive Temperature Index	In tenths of degrees Celsius, sum of the monthly average temperature of those months whose average temperature is higher than 0°C	scaled (degrees Celsius)
Tps2	Total temperature of the warmest bimonth of the summer quarter	Temperature (July+Sept)	degrees Celsius

Some indices listed below, for example m –the average minimum temperature of the coldest month, are not specified in the text but are needed to calculate the indices that are described in the text.

**Figure 2:**
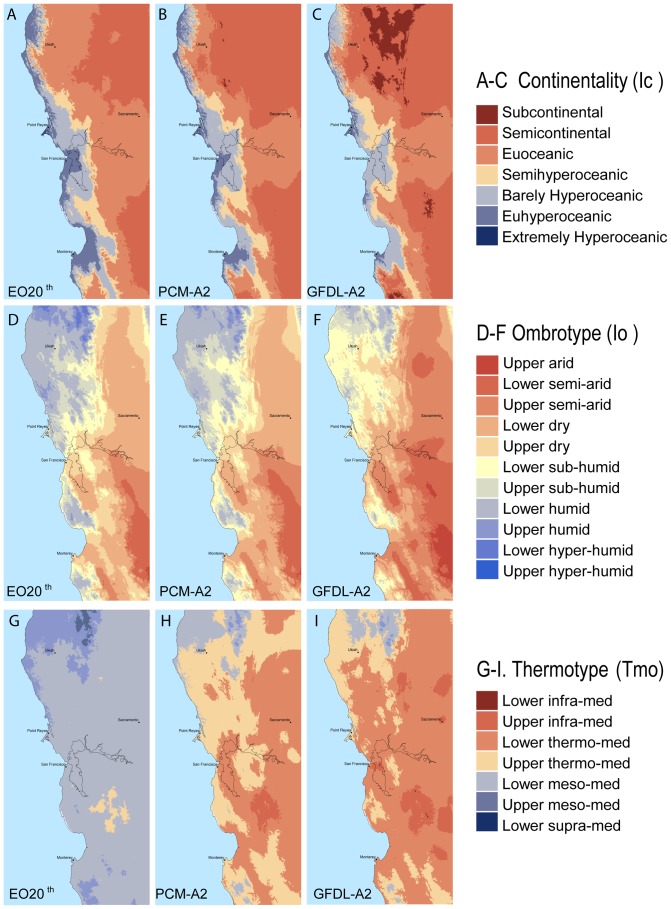
Distribution of three bioclimatic indices across the study area under three climatologies. ***A*** continentality during 1971–2000. ***B*** continentality projected for 2070–2099 under PCM-A2. ***C*** continentality projected for 2070–2099 under GFDL-A2. ***D*** ombrotype during 1971–2000. ***E*** ombrotype projected for 2070–2099 under PCM-A2. ***F*** ombrotype projected for 2070–2099 under GFDL-A2. ***G*** thermotype during 1971–2000. ***H*** thermotype projected for 2070–2099 under PCM-A2. ***I*** thermotype projected for 2070–2099 under GFDL-A2. Legend for thermotype classes abbreviates Mediterranean to “med.”

Continentality (Fig. 2A–C) is calculated as the range between the average temperatures of the warmest (Table 1, Tmax) and coldest months (Table 1, Tmin) of the year expressed in degrees Celsius. In the study area, the consistently warmest and coldest months the 100 years of the 20^th^ century are July and January respectively.

Ombrotype (Fig. 2D–F) is calculated as the ratio between the yearly positive precipitation in millimeters (Table 1, Pp) and the yearly positive temperature in degrees Celsius (Table 1, Tp). The yearly positive precipitation index is defined as the total average precipitation of those months whose average temperature is higher than 0°C. Yearly positive temperature is the sum of the monthly average temperature of those months whose average temperature is higher than 0°C. In some Mediterranean regions where the warmest months of the year are closer to the autumnal equinox rather than the summer solstice there is a need to use summer compensated ombrothermic indices to discriminate between isobioclimates at the edges of Mediterranean and Temperate macrobioclimates. None of the cells in the study area require this compensation.

Thermotypes (Fig. 2G-i) were assigned based on thresholds for the thermicity index (Table 1, It), compensated thermicity index (Table 1, Itc), and positive temperature index (Table 1, Tp). The thermicity index is calculated as a sum of the yearly average temperature, the average minimum temperature of the coldest month of the year, and the average maximum temperature of the coldest month of the year. In this study area, January is consistently the coldest month of the year. A compensated thermicity index is used when the continentality index value is above 18°C. When continentality is moderate (18.0<Ic< = 21.0) the Itc compensation value of 5 is added and when high (Ic>21.0) the compensation value is calculated as the sum of ((Ic -21)+15). The positive temperature index is included only when Ic>21 or It/Itc<120. For these cases the positive temperature index is derived as the sum of the monthly average temperature of those months whose average temperature is higher than 0°C. In the study area all months of the year have a monthly average temperature above 0°C.

monthly precipitation (mm), and minimum and maximum monthly air temperature (°C) products [34] that were spatially downscaled to 270-m grid cells using a modification [35] of the gradient plus inverse distance squared (GIDS) interpolation approach [36]. This interpolation scheme generates multiple regression equations for each month for each 4-km cell relative to each target 270-m grid cell using the relation of each climate variable to elevation and location. Assessments [35] comparing measured climate data from weather stations with the two interpolated products, the 4-km PRISM and downscaled 270-m climate grids, calculated higher R2 for the 270-m downscaled climate than PRISM for all climate values tested (Table 2). This reflects the capacity of higher precision grid cells to more closely reflect point data, all other things being equal. One advantage of this interpolation technique over kriging is that it does not require the assumption of stationarity of data and it incorporates spatial and temporal changes in adiabatic lapse rate and the influence on local climate.

**Table 2:** R-Squared values for downscaled climate parameter and PRISM data compared to weather station observations.

Climate Parameter	R^2^	
	4-km	270-m
Precipitation mm/month	0.6496	0.6497
Temperature (min) degrees C	0.866	0.8729
Temperature (max) degrees C	0.9147	0.9191

(Adapted from Figure 4. **Flint and Flint** ***Ecological Processes*** **2012** 1∶**2**).

Bioclimate indices were calculated for the two EO21st projections that were available from Thorne et al. [29]. These 270-m downscaled projections were developed on the basis of 12-km national maps of downscaled GCM output available at http://tenaya.ucsd.edu/wawona-m/downscaled/. Projected values for monthly precipitation (mm), and minimum and maximum monthly temperature (oC) were further downscaled to 270 meters in 3 steps [35]. The first step downscales the 12-km grids to 4-km using the GIDS approach to enable bias-correction to the PRISM grids. The second step applies the bias correction to adjust the mean and standard deviation to match those of the 1950–2000 record developed in PRISM. Once corrected, the 4-km grids are downscaled to 270-m grid using the GIDS approach [35].

To simplify the narrative describing the metrics, analyses, and results from this research only one emission scenario is reported. We selected the results from the A2-medium-high emission scenario because the CO_2_ emissions of both A-2 and the B1-low emission had already been well exceeded by 2012.

Changes in the spatial distribution of bioclimate index categories between the EO20^th^ and EO21^st^ periods were generated in using cell by cell raster ARCGIS processing. Change was quantified as the number of categories that differed between periods. For example, if a cell in the EO20^th^ period has the continentality category (Table 3) of extremely hyperoceanic (EXHO) and in the future period the cell was barely hyperoceanic (BHOC), it was assigned a change index of 2, if the cell had a continentality category of BHOC in both the EO20^th^ and future periods it was assigned a change of 0.

**Table 3:** Continentality index categories and range of values.

Codes		Continentality type	Value (It, Itc); CO*10
Alpha	Numeric		
Exho	1	Extremely Hyperoceanic	0–4
Euho	2	Euhyperoceanic	4.01–8
Bhoc	3	Barely Hyperoceanic	8.01–11
Seho	4	Semihyperoceanic	11.01–13
Euoc	5	Euoceanic	13.01–17
Seco	6	Semicontinental	17.01–21
Suco	7	Subcontinental	21.01–28

Isobioclimates were identified as unique categorical combinations of the three indices, continentality with 7 categories (Table 3), ombrotype with 12 categories (Table 4), and thermotype with 7 categories (Table 5). Novel isobioclimates are defined as those unique isobioclimates that are not present in the study area in the EO20^th^ period.

**Table 4:** Ombrotype index categories and range of values.

Codes		Ombrotype	Value (Io) mm/C
Alpha	Numeric		
UARI	5	Upper arid	0.6–1
LSAR	6	Lower semiarid	1.01–1.5
USAR	7	Upper semiarid	1.51–2
LDRY	8	Lower dry	2.01–2.8
UDRY	9	Upper dry	2.81–3.6
LSHU	10	Lower subhumid	3.61–4.8
USHU	11	Upper subhumid	4.81–6
LHUM	12	Lower humid	6.01–9
UHUM	13	Upper humid	9.01–12
LHHU	14	Lower hyperhumid	12.01–18
UHHU	15	Upper hyperhumid	18.01–24

**Table 5:** Thermotype index categories and range of values.

Codes		Thermotype	Value (It, Itc); CO*10	Value (Tp) used if Ic > = 21 or It, Itc<120
Alpha	Numeric			
Lsme	1	Lower supramediterranean	145–210	1200–1500
Umme	2	Upper mesomediterranean	211–280	1501–1825
Lmme	3	Lower mesomediterranean	281–350	1826–2150
Utme	4	Upper thermomediterranean	351–400	2151–2300
Ltme	5	Lower thermomediterranean	401–450	2301–2450
Uime	6	Upper inframediterranean	451–515	2451–2650
Lime	7	Lower inframediterranean	516–580	>2650

Associations between vegetation and bioclimate indices were developed with canonical correspondence analysis (CCA) implemented using CANOCO 4.5 [37]. Vegetation type abundance and distribution data from the statewide California Department of Forestry and Fire Protection, Fire and Resource Assessment Program Multi-Source Vegetation data layer [38] were clipped to the study area. Of the 77 statewide vegetation types 42 are found in the study area. Nineteen vegetation types were removed using a set of 3 criteria to generate a subgroup of 23 vegetation-types for the CCA ordination. Removal criteria included: 1) anthropogenic types not expected to change due primarily to climate such as urban and agriculture, 2) vegetation types dependent on local hydrology such as riparian and wetland, and 3) types classified as unknown shrubs and conifer. Abundance was defined as the percent cover of each vegetation type found in each isobioclimate with each isobioclimate treated as a plot. Each bioclimate index was treated as an environmental variable in the analysis: continentality, ombrotype, and thermotype.

## Results

All the cells in the study area in the EO20^th^ period and both projections of the EO21^st^ period had a summer ombrothermic index (Ios4) of less than 2 placing the entire study area in the Mediterranean macrobioclimate. Only the driest of the 7 major Mediterranean bioclimates, hyperdesertic Mediterranean, is not represented in the study in either the EO20^th^ or EO21^st^ periods.

**Figure 3:**
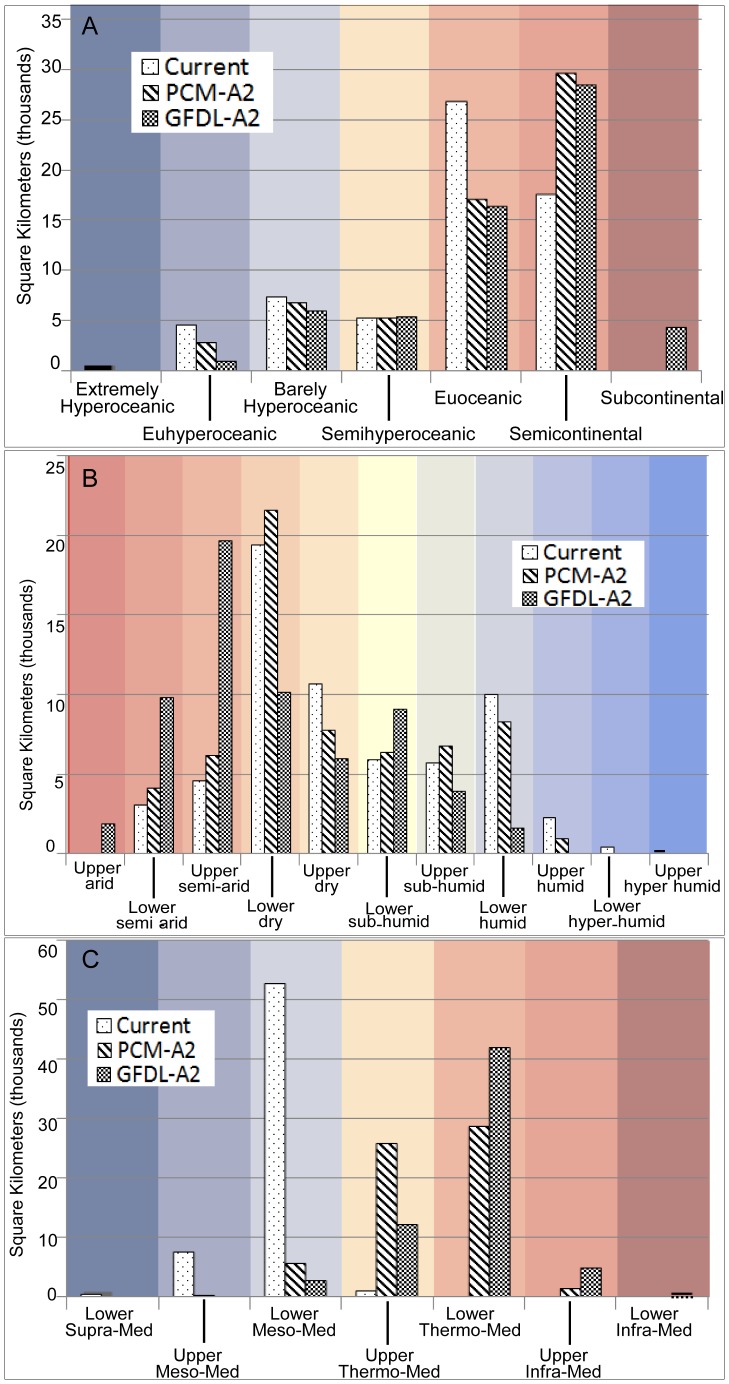
Areal comparison of three bioclimatic indices during the end of the 20^th^ century and two projections of end of the 21^st^ century. ***A*** continentality. ***B*** ombrotype. ***C*** thermotype. Thermotype classes use the abbreviation “med” for Mediterranean.

**Figure 4:**
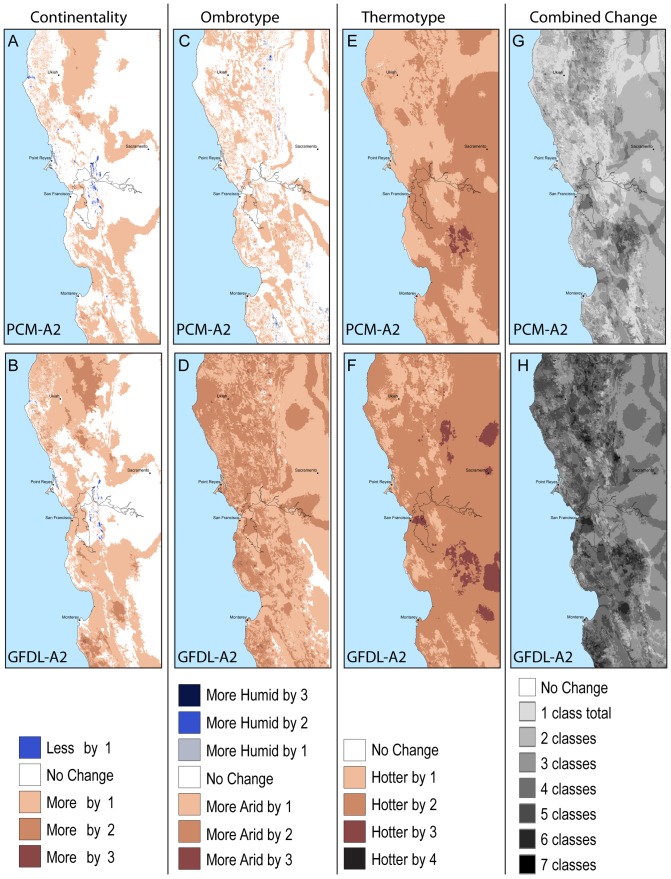
One hundred years of isobioclimate change. Numbers of classes of change in ***A*** continentality with the PCM-A2 projection. ***B*** continentality with the GFDL-A2. ***C*** ombrotype with the PCM-A2 projection. ***D*** ombrotype with the GFDL-A2 projection. E ombrotype with the PCM-A2 projection. ***E*** thermotype with the PCM-A2 projection. ***F*** thermotype with the GFDL-A2 projection. ***G*** Total number of classes of change summed from each bioclimate index in the PCM-A2 future. ***H*** Total number of classes of change summed from each bioclimate index in the PCM-A2 future.

The values for continentality (Ic) range from extremely hyperoceanic (EXHO) to semicontinental (SECO) in the EO20^th^ period (Table 3). In both modeled future projections an additional category of subcontinental appears (SUCO). In the EO20^th^ period the majority of the study area, about 27,000 km^2^, is in the euoceanic category (Fig. 2A and Fig. 3A) with an Ic value of 13°–17°C. At the end of the century under both the PCM-A2 and GFDL-A2 projections a majority, 30,000 km^2^ and 28,000 km^2^ respectively, transitions into the SECO range of 18–21°C (Fig. 2B, C, and Fig. 3A). Under the GFDL projection more area changes Ic values with 4400 km^2^ transitioning into a SUCO category predominantly in the north eastern portion of the study area (Fig. 2C) where topographic complexity and elevation is the highest (Fig. 1). Under the PCM-A2 projection the Point Reyes Peninsula remains extremely hyperoceanic. The marked coastal influence associated with the east-west connection of the bay-delta to the ocean is seen strongly in the EO20^th^ period and maintained in both future projections although much less in the GFDL-A2 projection (Fig. 4A and B). Continentality increases in areas of higher elevation and substantially so with GFDL-A2.

The lower dry (LDRY) ombrotype (Io) category covers the most area in both the EO21^st^ period and the PCM-A2 future at 19,000 km^2^ and 22,000 km^2^ respectively (Fig. 2E, f and Fig. 3B). The landscape of the GFDL-A2 future becomes much more arid with the majority of the landscape (20,000 km^2^) becoming upper semiarid (USAR). Most of the area in the GFDL-A2 projection shifts one or two categories drier with only a small area of no change in the southeastern portion of the landscape (Fig. 4D). This area of no change is in the driest of categories, upper arid (UARI), however had it become drier it would have brought in the regionally novel Io category, hyperarid. The distribution of Io change in both PCM-A2 and GFDL-A2 futures is closely associated with elevation with the lower elevations experiencing relatively less change than the higher elevations, except in the coastal area northwest of Ukiah under the GFDL-A2 future.

The dominant thermotype (Tmo) category in the study area, lower mesomediterranean (LMME), covers 53,000 km2 under EO20^th^ climate (Fig. 2G and 3C). Along the coast and in the northern coastal mountains the thermotypes are the cooler, upper mesomediterranean (UMME) and lower supramediterranean (LSME). The warmest Tmo, upper thermomediterranean (UTME), occurs under EO20^th^ conditions as a small patch in the interior, southeast of San Francisco. In both projections more than 99.7% of the landscape becomes warmer by 1–2 categories (Fig. 2H, 2I, 4E, and 4F) while in the GFDL-A2 scenario 84% of the landscape becomes warmer by 2 or more Tmo categories (Fig. 4F). The Point Reyes peninsula is one of the small areas that do not undergo a Tmo change but only under the future PCM-A2 projection (Fig. 4E).

**Figure 5:**
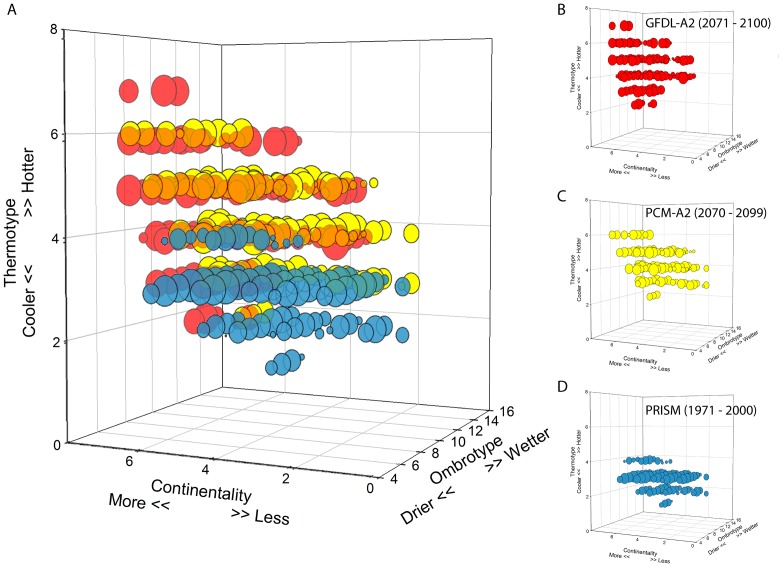
Unique Isobioclimates each located within a 3-dimensional cube of climate space. ***A*** The entire set of 195 isoclimate types representing climate conditions at the end of the 20^th^ century and two projections of the future, GFDL-A2 and PCM-A2. The size of the semi-transparent circles represents, on a logarithmic scale, the relative area for that unique isobioclimate across the landscape. Each temporal period/climate data source is represented by a different color, red = 2071–2100/GFDL-A2, yellow = 2070/PCM-A2 , and blue = 1971–2000/PRISM. Circles that appear orange are a result of red and yellow circles overlapping and represent isobioclimates that are common to both future projections. ***B*** 115 isobioclimate types of GFDL-A2 climate projections . ***C*** 108 isobioclimate types of PCM-A2 climate projections. ***D*** 83 isobioclimates of the end of 20^th^ century climate conditions.

**Figure 6:**
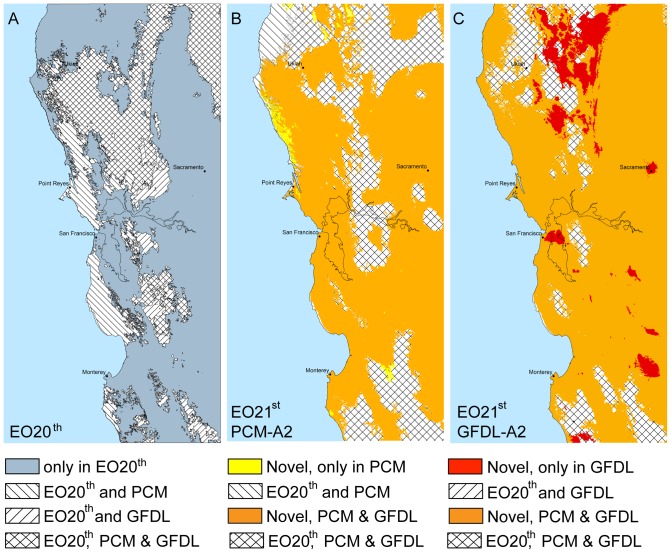
The study area seaprated into 4 categories based on the continuity of isobioclimates. ***A*** based on end of the 20^th^ century climate conditions, areas where isobioclimates dissapear in the future are coded blue, areas where the isobioclimates dissapear only under the GFDL projections (e.g. continue under PCM projection) are hatched to the right, areas where the isobioclimates dissapear only under the PCM projections (e.g. continue under GFDL projection) are hatched to the left, and areas where the isobioclimate continue under both projections are cross hatched. ***B*** novel and extant isobioclimates under the PCM-A2 climate projection. ***C*** novel and extant isobioclimates under the GFDL-A2 climate projection.

The three bioclimate indices (Io, Ic, Tmo) combined produce 83 unique isobioclimates in the EO20^th^ century, 108 in the PCM-A2 projection, and 115 in the GFDL-A2, for a total of 195 unique combinations (Table 6). When graphed in 3-dimensions (3-D) each unique isobioclimate can be represented by an x-y-z coordinate on a 3-D grid. Each climatological period has a distinct climate space that it occupies within the climate cube (Fig. 5). Isobioclimates found in the EO20^th^ and both future climate spaces show as overlapping points and represent isobioclimates extant into both projections of future conditions (Fig. 6A, B, and C). Some are present in the EO20^th^ but disappear in the future (Fig. 6A), and those that have no overlap are the regionally novel isobioclimates of the future (Fig. 6B and C). Isobioclimates unique to the PCM-A2 projections have a combination of the lowest values for continentality and middle thermotype values while those unique to GDFL-A2 are in the highest thermotype areas of the climate cube (Fig. 5).

**Table 6:** Number of isobioclimates common or unique in the EO21st climatology and two modeled projections of the EO22nd climatology.

Category	# of unique isobioclimates	Current	PCM-A2	GFDL-A2
EO20^th^ and found in both future projections	19	13,216	16,754	8,124
EO20^th^ and in PCM-A2	11	6,177	2,002	-
EO20^th^ and in GFDL-A2	2	3,176	-	5
EO20^th^ but not in either future projection	51	39,735	-	-
Novel found in PCM-A2 only	18	-	327	-
Novel found in GFDL-A2 only	34	-	-	4,494
Total	195	62,304	62,304	62,304

The EO20^th^ regional landscape has 51 isobioclimates covering close to 40,000 km2 that disappear in both future projections (Table 6, Fig. 5 and Fig. 6A). Eighteen novel isobioclimates are found exclusively in the PCM-A2 projection (Fig. 5) but occupy a small portion of the landscape 327 km^2^ (Fig. 6B). The 34 novel isobioclimates exclusive to the GFDL-A2 future occupy a larger area, 4,494 km2 and are concentrated mainly in the highest elevations northeast of Ukiah (Fig. 6C).

**Figure 7:**
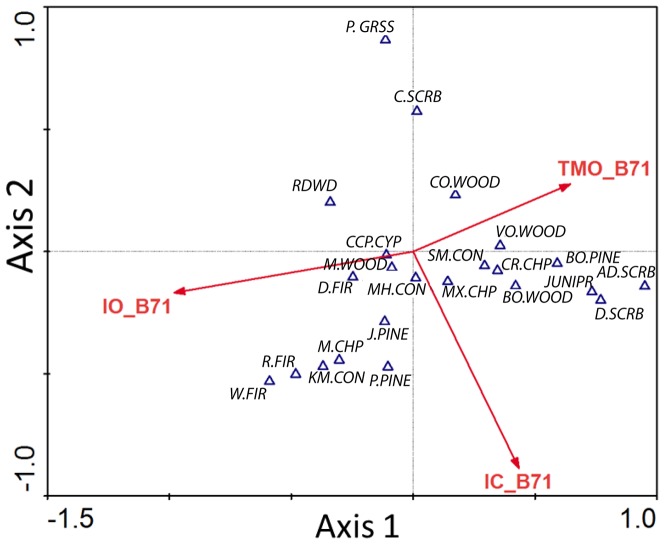
Canonical correspondence analysis (CCA) axes 1 and 2 biplots. The vegetation is ordinated with three 1971–2000 climate indices, continentality (IC_B71), ombrotype (IC_B71), and thermotype (TMO_B71). The eigenvalue vectors for the indices are superimposed on the CCA-biplot ordination to indicate relative influence of each index on species abundance and distribution. Vegetation codes: alkali desert scrub (AD.SCRB), blue oak-foothill pine (BO.PINE), blue oak woodland (BO.WOOD), coastal scrub (C.SCRB), closed-cone pine-cypress (CCP.CYP), coastal oak woodland (CO.WOOD), chamise-redshank chaparral (CR.CHP), Douglas-fir (D.FIR), desert scrub (D.SCRB), Jeffrey pine (J.PINE), juniper (JUNIPR), Klamath mixed conifer (KM.CON), montane chaparral (M.CHP), montane hardwood (M.WOOD), montane hardwood-conifer (MH.CON), mixed chaparral (MX.CHP), perennial grassland (P.GRSS), ponderosa pine (P.PINE), red fir (R.FIR), redwood (RDWD), sierran mixed conifer (SM.CON), valley oak woodland (VO.WOOD), white fir (W.FIR).

Each of the three indices represents a biologically relevant factor as demonstrated by the eigenvector lengths of the three environmental variables (Fig. 7) and the high correlation coefficients of the first three axes of the canonical correspondence analysis (CCA) of vegetation types as a function of the bioclimate index (Table 7). CCA is a constrained ordination, a matrix algebra based eigenanalysis [39] that measures the strength of the relationship between abundance (in this case dominant vegetation type) and environmental factors (in this case three bioclimate indices). The correlation coefficients and eigenvalues of the three indices and axes suggest that ombrotype is the strongest determinant of vegetation distribution followed by continentality and thermotype. The plot of the first two CCA axes (Fig. 7) shows that Axis 1 is dominated by ombrotype; for example redwood and Douglas-fir are to the left (wetter), montane hardwoods and mixed chaparral occupy the center, and blue-oak, valley oak, and coast live oak woodlands are to the right (drier), with juniper and desert scrub occupying the driest ombrotypes at the far right. The second axis is dominated by continentality - coastal scrub and perennial grasslands occupy the most oceanic areas at the top. Redwood tends toward more oceanic climates than Douglas-fir, while they occupy similar ombrotypes. Similarly, coast live oak and blue-oak woodlands are differentiated by continentality. Although montane conifer types (including Jeffery pine, ponderosa pine, red fir and white fir) are relatively rare in the region (small areas in the northern mountains) they extend into the lower left quadrant (wet ombrotypes, more continental) in a reasonable sequence. These relationships are well known botanically [40], [41] and give added confidence to the use of the bioclimate indices.

**Table 7:** Regression/canonical coefficients for ombrotype (IO_c71), continentality (IC_C71), and thermotype (TMO_C71) under EO21^st^ climatology for 3 ordination axes.

	EIGENVALUE	AXIS 1	AXIS 2	AXIS 3
CODE	INDEX			
IO_B1	OMBROTYPE	−0.8803	−0.2700	−0.9009
IC_B1	CONTINENTALITY	0.1932	−0.9954	−0.2105
TMO_B1	THERMOTYPE	0.0824	0.2589	−1.2281

## Discussion

The Rivas-Martinez classification successfully captured the high spatial climatic variability in the California coast range and allowed for delineation of isobioclimates at a fine spatial resolution. These isobioclimates are correlated with the proportions of vegetation-types in well-known patterns and provide a template for considering potential shifts in vegetation.

Seasonal and diurnal temperature fluctuations that are strongly influenced by ocean-land-atmosphere processes, such as attenuation of temperature extremes in coastal areas by fog, are a measure of the maritime coastal influence. This coastal effect, reducing the difference between maximum and minimum seasonal and daily temperatures is captured by the continentality index. In the study area the strong bay-delta signature, noticeable at the coastal opening of the Golden Gate, extends east. Marine stratocumulus and fog enter into the study area lowering the maximum temperatures during the day, increasing the minimum temperatures at night, and muting seasonal temperature swings. In both future projections, especially in GFDL-A2 there is a shift toward more continentality. The vegetation ordination shows perennial grassland, coastal scrub, redwood, and coastal oak woodland distributed toward the lower end of Ic (Fig. 7) suggesting these communities types would contract under GFDL-A2 conditions. Improving modeling of ocean-atmosphere-land dynamics would help to better predict potential future changes in fog and therefore improve forecasts of continentality and the impact on vegetation types associated with foggier coastal areas.

The thermotype landscape shows a greater patchiness between types in future projections and has the greater deviation from EO20^th^ conditions as measured by the number of thermotype class difference (Fig. 2G–I and 3E–F). Many future isobioclimate types consist of existing combinations of continentality and ombrotype but become regionally novel isobioclimate types when the thermotype index is added to the combination. The thermotype index has the weakest correlation coefficient in the CCA ordination. Further analysis could clarify if this is due to insufficient categories for successfully quantifying the thermal impact on vegetation or greater thermal tolerance in species adapted to Mediterranean climate regimes.

Unlike the widespread view that vegetation envelopes will move upslope and north into higher latitudes, in this region the cooler refugia are west toward the coast. These results are consistent with Loarie et al. 2008 that showed coastal areas having the highest potential for maintaining cooler mesic habitats. Indeed the west coast of North America has been a biogeographic refuge over geologic time because of the moderating influence of the Pacific Ocean [42], [43], [44], [45]. Global climate-driven changes in offshore currents and coastal upwelling, such as have been documented in the paleo-record [46], [47], as well as more recently [48], highlight the influence of ocean conditions on terrestrial climate.

The high correlation between biota and isobioclimates can be used to simplify complex distributional patterns. The categorical breaks along each of the bioclimate indices represent thresholds that have been defined because they are useful for distinguishing vegetation patterns on the landscape. Climate shifts that jump across several bioclimate categories represent multiple quanta of change across the landscape. Simple metrics of composite change summed up from each dimension suggest a landscape with sufficient complexity to harbor potential ecosystem resilience and could be used to further test hypotheses of species persistence [49]. Identifying areas of greatest expected isobioclimate change helps to identify areas of greatest vulnerability especially when different models project the same locations to change the most (Fig. 4). This geolocational intelligence can assist land managers to identify specific refugia, prioritize adaptive management efforts, and target lands for acquisition.

Future work on isobioclimates such as developing additional metrics to simplify maps of total change (Fig. 4G and 4H) could help to sharpen our understanding of vulnerability as it relates to different dimensions of climate change. Improved metrics to compare change of isobioclimate across climate space could be used to test hypotheses of relative resilience or determine climate dimensions of greatest impact. For example, some areas of greatest regional novelty such as “lower hyperhumid – hyperoceanic – upper thermomed” conditions in the PCM-A2 projection (small isolated yellow circle in fig. 5 with an isobioclimate climate space location of X = 11, Y = 1, Z = 4) cover relatively small areas (Fig. 6B). Yet their impact could be ecologically quite large if this particular isobioclimate provides a harbor for pathogens that allows them to get a regional foothold. Isobioclimates represent climate conditions averaged over at least 30 years which means that some years will be more wet or dry than others. The expansion and contraction of pathogenic, or invasive, populations during these years of extreme conditions is enhanced if refugia remain somewhere on the landscape. Understanding the geolocational identity of these foci can help to better understand the spread of propagules of interest.

**Figure 8:**
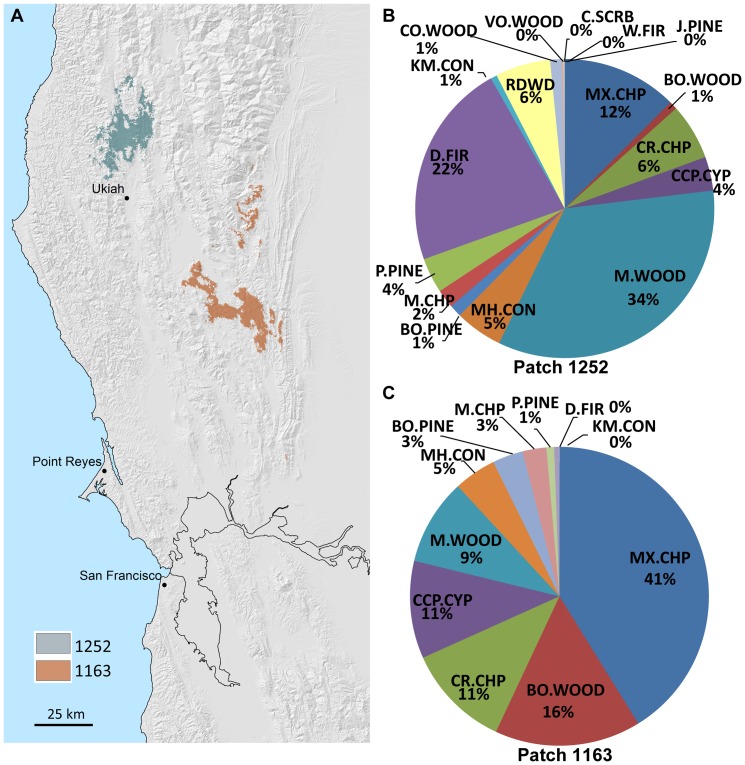
Isobioclimate patch analogs. ***A*** Map of two locations with different isobioclimates, 1252 and 1163 during the end of 20th century climate condition. Under the climate conditions of the PCM-A2 projection, patch 1252 (the blue area in the north) will have the same isobioclimate as patch 1163 (the red area to the southeast) does at the end of the 20th century. ***B*** dominant shrub and tree abundance in patch 1252 based on end of 20th century California Fire and Resource Assessment vegetation maps. ***C*** dominant shrub and tree abundance in patch 1163 from same vegetation map.

Current bioclimate analogs can also be used to identify areas in the current landscape that contain conditions similar to those that might be expected in the future. For example in Fig. 8A the patch identified as isobioclimate 1252 currently has lower humid – euoceanic – upper mesomediterranean climate conditions. In the PCM-A2 future it is projected to become isobioclimate 1163 with upper subhumid – semicontinental – lower mesomediterranean conditions. The current, e.g. EO20^th^, isobioclimate, 1252, supports primarily montane hardwood, Douglas fir, mixed chaparral, redwood, chamise chaparral, and small percentages of other vegetation types (Fig. 8B). Under the PCM-A2 scenario, isobioclimate patch 1252 will become patch analog 1163, which is currently found to the southeast and is a mix of mixed chaparral, blue oak woodland, closed-cone pine cypress, chamise chaparral, montane hardwood, and small percentages of 6 other vegetation types (Fig. 8C). Note that every vegetation type in patch 1163 is already present in patch 1252 suggesting that long distance dispersal, at least at the dominant species level, is not necessary. Therefore, vegetation change will primarily be a local rearrangement of species already in place. However, several dominant species, in particular redwood, are not currently found in patch 1163, and these vegetation types would be expected to be lost when this location transitions into isobioclimate 1252 if future projections hold.

Adaptive management strategies implemented by natural resource managers could include identifying and protecting small local patches of the more arid vegetation/species that provide foci for spread. For example, the 1% blue oak woodland found in patch 1252 serves as the nucleus for expansion to the relative abundance of 16% currently found in patch 1163. Another strategy, albeit more controversial, is managed translocation of species from analog patches to enhance habitat value. Very fine scale topoclimate variability becomes an important factor because short distance dispersal, such as from the other side of a canyon, may provide propagules for community recombination. This suggests differentiating the landscape into areas where the velocity of climate change is more or less important depending on the dispersal abilities of species [50], [51], [52] and taking advantage of higher resolution elevation data to better incorporate topoclimatic variation. Developing adaptive management strategies will be further challenged by ecosystem processes that are stimulated or exacerbated by increasing temperatures or changing precipitation patterns such as wildfires [53] and the increased photosynthetic efficiency in response to increasing levels of CO_2_ [54], [55].

The geography of climate change is complex and multi-scaled [56]. The relationship between climate and vegetation mosaics in California mountains is a fine scale process driven by topoclimatic effects (solar radiation, cold air pooling) and below the scale of this analysis. The next step but beyond the scope of this study, is to investigate analog patterns of bioclimatic -vegetation relationships using higher resolution isobioclimate and vegetation data across larger areas using CCA or other statistical techniques [57], [58]. Extending high resolution isobioclimate mapping efforts to larger areas, will affect what is labeled a novel isobioclimate. Isobioclimate novelty is related to the spatial scale of analysis. Isobioclimates that are novel on a regional scale may lose their novelty at the state-wide scale if analogs exist for them at the state-wide scale. The concept of regionally novel isobioclimates is none-the-less important for ecological conservation purposes. Conceptually it is similar to the distinction made between local and global rarity of plants species. These distinctions are important for conservation and protection of rare species and arise in part from research into the process of extinction and geographic fragmentation.

The analyses in this paper characterize the landscape in ways that can be used for land management decisions. The biologically relevant categories that define individual isobioclimate units facilitate their use as analytic units to explore change across the landscape. Isobioclimate analogs provide a framework to generate hypotheses and forecasts of shifts in vegetation community structure in a response to climate change. Implementing a worldwide bioclimatic classification system at regional to local scales provides a multi-scale framework for investigating the response of biotic systems to climate change.
